# Glucocorticoids with different chemical structures but similar glucocorticoid receptor potency regulate subsets of common and unique genes in human trabecular meshwork cells

**DOI:** 10.1186/1755-8794-2-58

**Published:** 2009-09-10

**Authors:** Alissar Nehmé, Edward K Lobenhofer, W Daniel Stamer, Jeffrey L Edelman

**Affiliations:** 1 Department of Biological Sciences, Allergan, Inc., Irvine, CA 92612, USA; 2 Cogenics™, A Division of Clinical Data®, Morrisville, NC 27560, USA; 3 Department of Ophthalmology and Vision Science, University of Arizona, Tucson, AZ 85724, USA; 4 Current address : Amgen, Inc., Thousand Oaks, CA 93120, USA

## Abstract

**Background:**

In addition to their well-documented ocular therapeutic effects, glucocorticoids (GCs) can cause sight-threatening side-effects including ocular hypertension presumably via morphological and biochemical changes in trabecular meshwork (TM) cells. In the present study, we directly compared the glucocorticoid receptor (GR) potency for dexamethasone (DEX), fluocinolone acetonide (FA) and triamcinolone acetonide (TA), examined the expression of known GRα and GRβ isoforms, and used gene expression microarrays to compare the effects of DEX, FA, and TA on the complete transcriptome in two primary human TM cell lines.

**Methods:**

GR binding affinity for DEX, FA, and TA was measured by a cell-free competitive radio-labeled GR binding assay. GR-mediated transcriptional activity was assessed using the GeneBLAzer beta-lactamase reporter gene assay. Levels of GRα and GRβ isoforms were assessed by Western blot. Total RNA was extracted from TM 86 and TM 93 cells treated with 1 μM DEX, FA, or TA for 24 hr and used for microarray gene expression analysis. The microarray experiments were repeated three times. Differentially expressed genes were identified by Rosetta Resolver Gene Expression Analysis System.

**Results:**

The GR binding affinity (IC_50_) for DEX, FA, and TA was 5.4, 2.0, and 1.5 nM, respectively. These values are similar to the GR transactivation EC_50 _of 3.0, 0.7, and 1.5 nM for DEX, FA, and TA, respectively. All four GRα translational isoforms (A-D) were expressed in TM 86 and TM 93 total cell lysates, however, the C and D isoforms were more highly expressed relative to A and B. All four GRβ isoforms (A-D) were also detected in TM cells, although GRβ-D isoform expression was lower compared to that of the A, B, or C isoforms. Microarray analysis revealed 1,968 and 1,150 genes commonly regulated by DEX, FA, and TA in TM 86 and TM 93, respectively. These genes included RGC32, OCA2, ANGPTL7, MYOC, FKBP5, SAA1 and ZBTB16. In addition, each GC specifically regulated a unique set of genes in both TM cell lines. Using Ingenuity Pathway Analysis (IPA) software, analysis of the data from TM 86 cells showed that DEX significantly regulated transcripts associated with RNA post-transcriptional modifications, whereas FA and TA modulated genes involved in lipid metabolism and cell morphology, respectively. In TM 93 cells, DEX significantly regulated genes implicated in histone methylation, whereas FA and TA altered genes associated with cell cycle and cell adhesion, respectively.

**Conclusion:**

Human trabecular meshwork cells in culture express all known GRα and GRβ translational isoforms, and GCs with similar potency but subtly different chemical structure are capable of regulating common and unique gene subsets and presumably biologic responses in these cells. These GC structure-dependent effects appear to be TM cell-lineage dependent.

## Background

Glucocorticoid (GC) therapy can lead to the development of glaucomatous ocular hypertension and secondary open-angle glaucoma that is clinically similar to primary open-angle glaucoma [1]. The elevated intraocular pressure (IOP) is due to increased aqueous humor outflow resistance and is associated with morphological and biochemical changes in the trabecular meshwork (TM) [2]. These changes are associated with increased deposition of extracellular matrix material in the outflow pathway [3], which may be due, in part, to an inhibition of TM phagocytosis [4], decreased extracellular activity of stromelysin and tissue plasminogen activator [5], alteration of the actin cytoskeleton [6] and formation of intercellular junctions [7]. Most of the effects of GCs on TM cells and tissues are likely due to GC-mediated TM cell gene expression, including the induction of myocilin, serum amyloid A1, α A-crystallin, insulin growth factor binding protein 2, growth arrest-specific 1 and other genes [8-13]. It is currently unclear which genes or combinations of genes are modulated by GCs and ultimately lead to increased IOP.

The biological actions of glucocorticoids are mediated through the cytoplasmic glucocorticoid receptor (GR), which belongs to the nuclear receptor subfamily that includes receptors for mineralocorticoids, estrogen and thyroid hormones, retinoic acid, and vitamin D [14]. Upon hormone binding, the activated ligand-bound receptor translocates into the nucleus and binds as a homodimer to glucocorticoid response elements within the promoter region of target genes. The GR can positively or negatively regulate gene expression, depending on the response element sequence and promoter context. The GR also modulates gene expression, independent of glucocorticoid response elements, by physically interacting with other transcription factors (e.g., activating protein AP-1 and nuclear factor NF-κB) [15].

GRα and GRβ are the two major splice variants of GR as a result of alternative splicing. The GRβ isoform acts as a natural dominant negative inhibitor of GRα-induced transactivation of glucocorticoid-responsive genes [16]. Amino acid sequence analysis revealed that GRα and GRβ isoforms are identical from the amino terminus to amino acid 727 but diverge beyond this position, with GRα having an additional 50 amino acids and GRβ having an additional, non homologous, 15 amino acids. The expression of both GRα and GRβ was reported in cultured human TM cells [17,18].

Recent evidence indicates that at least eight different GRα or GRβ N-terminal isoforms are generated from one single GR gene by alternative translation initiation [19]. For GRα, receptor isoforms are designated GRα-A, -B, -C1, -C2, -C3, -D1, -D2 and -D3 [20]. The GRα-A isoform is the full-length receptor containing amino acids 1-777. The other GRα isoforms have shortened N termini. The apparent molecular weights for GRα-A, -B, -C and -D are 94, 91, 82-84 and 53-56 KDa, respectively. All GRα isoforms are functional receptors and contain the identical intact ligand-binding domain that binds GCs. The GRα isoforms transcriptionally regulate common and unique sets of genes within the context of a single cell type [20]. Furthermore, the tissue expression patterns of GRα translational isoforms have been determined in both rats and mice and the levels of the GRα isoforms differ widely among tissues [20]. Thus, the unique transcriptional activities and distinct tissue-specific distribution patterns of GRα isoforms could provide a novel mechanism for tissue-specific glucocorticoid responses. Further studies are needed to confirm this in human tissues. For GRβ, receptor isoforms are designated GRβ-A, -B, -C1, -C2, -C3, -D1, -D2 and -D3 [19]. The GRβ-A isoform is the full-length receptor containing amino acids 1-742 with an apparent molecular weight of 90 KDa. The other GRβ isoforms have shortened N termini. Although little is known about the transcriptional activity and tissue distribution of GRβ isoforms, Fruchter et al demonstrated that the potency of the dominant negative effect of GRβ on GRα-induced transactivation depends on both the type and the dose of the synthetic glucocorticoids in use [16].

Recently, Schaaf et al showed that the intranuclear distribution and mobility of the GR is highly dependent on the chemical structure of the glucocorticoid with which it is associated [21]. Some ligands, especially high-affinity synthetic ligands like dexamethasone and triamcinolone acetonide, induce a highly punctate GC-GRα distribution organized in discrete domains of high receptor concentration. In contrast, other ligands, mainly naturally occurring low-affinity ligands like cortisone and cortexolone, induce a more homogeneous, although still not entirely random, distribution. More importantly, structure-function analysis revealed that the 9-fluoro and 17-hydroxy groups on the steroid significantly impact nuclear receptor distribution. The effects of GC-mediated GR nuclear distribution and mobility patterns on GR-dependent transcriptional activity are still unclear.

Microarray technology provides a comprehensive, rapid and efficient method for large scale profiling of gene expression changes in biological samples (e.g., treatment versus control, disease versus normal). The advantages of DNA microarray technology include the ability to analyze expression patterns of thousands of genes simultaneously. Other advantages include the ability to characterize relationships between genes and the changes in biological processes such as disease states, developmental stages and responses to drugs [22,23]. More importantly, Canales et al recently showed that results generated from five different microarray platforms correlated exceedingly well with real-time quantitative polymerase chain reaction (RT-PCR), as well as other non-microarray-based approaches to determine transcript abundance [24].

In this study, using two primary human TM cell lines isolated from either a newborn or an adult donor, we examined the distribution of GRα and GRβ isoforms and determined global gene expression profiles after treatment with 1 μM of three potent GR agonists (dexamethasone, fluocinolone acetonide and triamcinolone acetonide). TM 86 and TM 93 expressed all known GRα and GRβ isoforms as determined by Western blot. The gene expression profiles and Ingenuity Pathway Analysis demonstrated that each of the three GCs regulated a common and unique subset of genes that is associated with a cell lineage-dependent specific signaling pathway.

## Methods

### Cell culture and glucocorticoid treatment conditions

Human primary cultures of trabecular meshwork cells, TM 86 and TM 93, were established and characterized from 3-month- and 35-year-old human eye donors, respectively, according to original methods [25]. Cells were maintained in low glucose Dulbecco's Modified Eagle Medium (DMEM, Invitrogen, Carlsbad, CA) supplemented with 10% (vol/vol) FBS (American Type Culture Collection, Manassas, VA), 1% (vol/vol) penicillin and streptomycin (Invitrogen). Cells from the human HeLa cervical adenocarcinoma cell line (American Type Culture Collection) were maintained in α-MEM medium supplemented with 10% (v/v) FBS, 1% (v/v) penicillin and streptomycin. All cells were used within passages 3 to 6.

For treatment with DEX, FA, or TA, TM cells were grown in 100-mm tissue culture plates (BD Labware, Bedford, MA) in DMEM complete medium until reaching 100% confluence. Cells were maintained for one week with medium renewal every other day. The day before the experiment, confluent TM were washed once with phosphate-buffered saline (PBS) and then the cells were maintained in 1% FBS-containing medium with antibiotics. Cells were treated with 1 μM of DEX, FA, or TA for an additional 24 hr. Control cells were treated with 0.1%(v/v) dimethylsulfoxide (DMSO). At the end of GC treatment, supernatant was discarded and the cell monolayer was kept frozen at -80°C for subsequent total RNA extraction and gene expression profiling.

### Glucocoroticoid receptor binding affinity

GR binding affinity assay was performed at Cerep Inc. (Celle l'Evescault, France), according to an established method [26]. Brifely, fractions of IM-9 human B lymphoblast cell cytosol (300 μg proteins) are incubated for 6 h at 4°C with 1.5 nM [3H]dexamethasone in the absence or presence of DEX, FA, or TA in a buffer containing 10 mM Tris ethanesulfonic acid-NaOH (pH 7.4), 1 mM EDTA, 10 mM Na2MoO4, 20 mM β-mercaptoethanol and 10% glycerol. Nonspecific binding is determined in the presence of 10 μM triamcinolone. Following incubation, the samples are filtered rapidly under vacuum through glass fiber filters (GF/B, Packard) presoaked with 0.3% Poly(ethyleneimine) and rinsed several times with ice-cold 50 mM Tris-HCl using a 96-sample cell harvester (Unifilter, Packard). The filters are dried and counted for radioactivity in a scintillation counter (Topcount, Packard) using a scintillation cocktail (Microscint 0, Packard). The results are expressed as percent inhibition of the control radioligand specific binding. The IC_50 _values (concentration causing half-maximal inhibition of control specific binding) and Hill coefficients (*nH*) were determined by non-linear regression analysis of the competition curves generated with mean replicate values using Hill equation curve fitting (Y = D + [(A - D)/(1 + (C/C_50_)^nH^)], where Y = specific binding, D = minimum specific binding, A = maximum specific binding, C = compound concentration, C_50 _= IC_50_, and nH = slope factor). This analysis was performed using the Hill software (Cerep) and validated by comparison with data generated by the commercial software SigmaPlot^® ^4.0 for Windows^® ^(^© ^1997 by SPSS Inc.). The inhibition constants (K_i_) were calculated using the Cheng Prusoff equation (K_i _= IC_50_/(1+(L/K_D_)), where L = concentration of radioligand in the assay, and K_D _= affinity of the radioligand for the receptor). The standard reference compound is DEX, which is tested in each experiment at several concentrations to obtain a competition curve from which an IC_50 _is calculated. The GR binding assay was performed in duplicate.

### Glucocoroticoid receptor-mediated transactivation

GR transactivation activity was assessed by the Invitrogen SelectScreen Profiling Service (Madison, WI), using Invitrogen GeneBLAzer beta-lactamase reporter gene technology [27]. Profiling was performed using human HeLa cells stably transfected with an expression construct containing β-lactamase cDNA under the control of the MMTV response element previously identified as a GR response element (MMTV-bla HeLa CellSensor^®^). The MMTV-bla HeLa cells were plated in 384-well plates in 32 μl assay medium (OPTIMEM + 1% charcoal/dextran stripped FBS) at a density of 10,000 cells/well and incubated overnight. After addition of 8 μl of a 5-fold concentrated compound solution (DEX, FA, or TA) in assay medium, the cells were incubated for additional 5 hr. The 5 hr time point was chosen based on preliminary results showing that the optimal DEX-mediated transcriptional activation of GR occurred between 5 and 16 hr. The β-lactamase activity of each sample was determined by adding 8 μl of 6-fold concentrated LiveBLAzer™ substrate solution to the samples followed by two hours of incubation at room temperature in a Tecan Safire2 plate reader using the appropriate LiveBLAzer™ filter settings. GC treatment was performed in 15-point dose response using a three-fold dilution series starting with a maximum compound concentration of 10 μM. Each data point represents a triplicate sample (n = 3). Assay reproducibility was determined by calculating Z' values for untreated vs. maximum stimulation. The GeneBLAzer assay was performed twice.

### Distribution of glucocoroticoid receptor isoforms in TM 86 and TM 93 cells

TM cells were grown in 100-mm tissue culture plates (BD Labware) in DMEM complete medium until reaching 100% confluence. Cells were then maintained for one week with medium renewal every other day. Human HeLa cells were used as a positive control in these experiments. The day before the experiment, confluent TM and HeLa cells were washed once with phosphate-buffered saline (PBS) and stepped to 1% FBS-containing medium with antibiotics. After 24 hr, cells were washed twice with ice-cold PBS and lysed in 400 μL radioimmuno-precipitation assay buffer (50 mM Tris-HCl [pH 7.4], 150 mM NaCl, 0.25% deoxycholic acid, 1% NP-40, 1 mM EDTA; Upstate Biotech, Lake Placid, NY), supplemented with 0.2% sodium dodecyl sulfate (SDS) and a 1% (vol/vol) cocktail of protease inhibitors, serine-threonine phosphatase inhibitors, and tyrosine phosphatase inhibitors (Sigma-Aldrich, Saint Louis, MO). After 30 min incubation on ice, the lysates were centrifuged at 14,000 rpm for 20 min at 4°C. Supernatants were transferred to clean microfuge tubes, and the total protein concentration of each sample was measured with a bicinchoninic acid protein assay (Pierce Biotechnology, Rockford, IL). Cell lysates (45 μg protein) in SDS sample buffer were separated on 7% Tris-acetate gel by SDS-polyacrylamide gel electrophoresis and transferred to nitrocellulose membranes. Four GRα isoforms were detected by a specific polyclonal antibody anti-GRα (PA1-516) from ABR Affinity BioReagents (Golden, CO), whose epitope corresponds to the specific GRα peptide segment aa 755-771 [20]. Four GRβ isoforms were detected by a specific polyclonal antibody anti-GRβ (PA3-514) from ABR Affinity BioReagents, whose epitope corresponds to the specific GRβ peptide segment aa 728-742 [19]. Protein bands were visualized with a chemiluminescence detection kit (Invitrogen). Three independent cell lysate samples were tested per cell line for each antibody.

### RNA isolation and gene expression profiling

RNA isolation and gene expression profiling were performed at Cogenics™, A Division of Clinical Data^® ^(Morrisville, NC), using methods that have been previously described [28-31]. Briefly, RNA was isolated from flash frozen cell culture plates using RNeasy Mini columns (Qiagen, Valencia, CA) according to the manufacturer's protocol. There were three biological replicates per treatment condition. The quantity and purity of the extracted RNA was evaluated using a NanoDrop ND-1000 spectrophotometer (Nanodrop Technologies, Wilmington, DE, USA) and its integrity measured using an Agilent Bioanalyzer. For microarray hybridizations, 500 ng of total RNA from each RNA sample was amplified and labeled with a fluorescent dye (Cy3) using the Low RNA Input Linear Amplification Labeling kit (Agilent Technologies, Palo Alto, CA, USA) following the manufacturer's protocol. The amount and quality of the fluorescently labeled cRNA was assessed using a NanoDrop ND-1000 spectrophotometer and an Agilent Bioanalyzer. An equal amount of Cy3-labeled cRNA (1.6 μg) was hybridized to the Agilent Human Whole Genome Oligo Microarray (Agilent Technologies) for 17 hours, prior to washing and scanning. Data were extracted from scanned images using Agilent's Feature Extraction Software (Agilent Technologies) using default settings. Gene expression data were loaded into the Rosetta Resolver^® ^Gene Expression Analysis System version 7.1.0.1.11. Data from three biological replicate hybridizations were combined using an error-weighted average and the following criteria was used to identify differentially expressed transcripts: a log ratio *p*-value < 0.001, and a log(10) intensity measurement > -1.8. The full complement of microarray data has been deposited in the NIH/NLM Gene Expression Omnibus (GEO accession # GSE16643) [32].

Gene ontology enrichment analysis was performed on lists of differentially expressed transcripts using the High-Throughput GoMiner integrative gene ontology tool with a significance threshold of an enrichment *p*-value < 0.01 [33,34]. The lists of differentially expressed genes were also applied to global functional, network and canonical pathway analyses using Ingenuity Pathway Analysis (Ingenuity^® ^Systems, Redwood City, CA). In order to identify the most significant results in IPA's analyses, the Benjamini-Hochberg multiple-testing corrected p-value was used with a significance threshold of 0.05.

## Results

### Glucocorticoid receptor binding affinity

The GR binding affinity for DEX, FA and TA has never been directly compared in one cell type. Moreover, a wide range of GR binding affinities have been reported in the literature for each of these three GCs [35]. Therefore, we sought to directly compare the GR binding affinity *in vitro *using a GR radiolabeled competitive binding assay (Cerep, France). The results are shown in Table 1. The IC_50 _values for DEX, FA, and TA were 5.4, 2.0, and 1.5 nM, respectively. These results suggest that the GR binding affinities for these three drugs are roughly equivalent. Interestingly, the GR binding affnitiy of 5 nM for DEX in IM-9 cells is consistent with previously reported cellular binding affinity of DEX in primary human TM cells [18]. The distribution of GRα isoforms in human IM-9 cells used in the GR binding affinity assay is unknown. Since different GRα isoforms might exhibit different binding affinities for each GC, the observed GR binding affinity is likely an aggregate affinity generated by multiple GR isoforms.

**Table 1:** Glucocorticoid receptor binding affinity for dexamethasone, fluocinolone acetonide, and triamcinolone acetonide.

**Ligand**	**IC_50_** **(nM)**	**K_i_** **(nM)**	***n*_*H*_**
Dexamethasone	5.36 ± 1.13	2.68 ± 0.58	1.06 ± 0.18

Fluocinolone acetonide	2.00 ± 0.42	0.97 ± 0.17	1.10 ± 0.00

Triamcinolone acetonide	1.45 ± 0.50	0.72 ± 0.24	1.05 ± 0.07

IC_50_, K_i_, and *n*_*H *_values were determined using a glucocorticoid receptor radiolabeled competition binding assay as described in "Materials and methods" section.

Data shown for dexamethasone are averages of fourteen independent experiments performed in triplicate ± SD.

Data shown for fluocinolone acetonide and triamcinolone acetonide are averages of two independent experiments performed in triplicate ± SD.

### Glucocorticoid receptor transactivation potency

**Figure 1:**
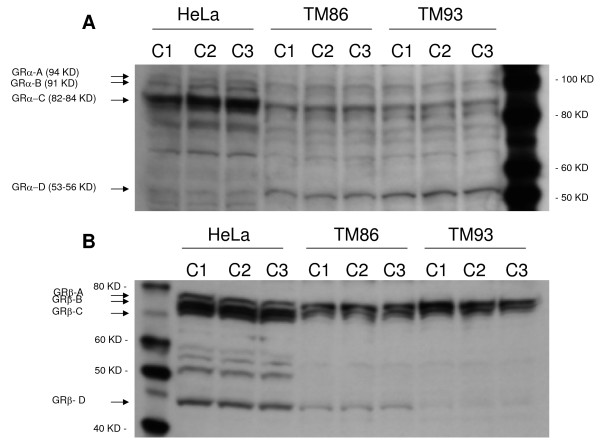
**Western blot analysis of the GRα (A) and GRβ (B) isoforms in human TM86 and TM93**. Blots resolving 45 μg of total cell lysates from HeLa, TM 86, or TM 93 cells detected with anti-GRα antibody (PA1-516) or anti-GRβ antibody (PA3-514) are shown. Three independent cell lysates were analyzed per cell line. Additional bands in between GR-C and GR-D isoforms may be degradation products or cell-specific isoforms.

**Figure 2:**
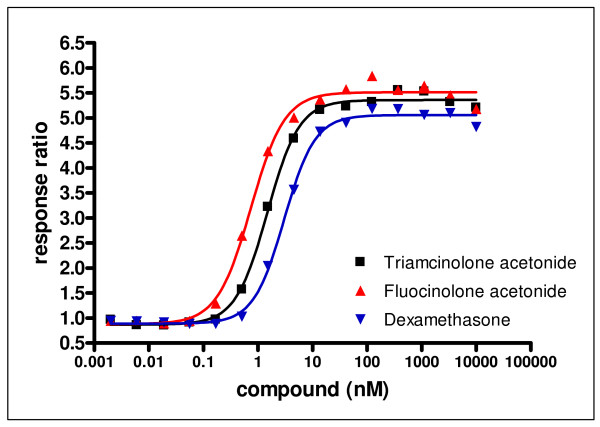
**Activation of glucocorticoid receptor in response to dexamethasone, fluocinolone acetonide and triamcinolone acetonide**. MMTV-bla Hela CellSensor^® ^cells were treated with GC for 5 hr. β-lactamase reporter gene activity was assessed by adding the LiveBLAzer substrate solution for 2 hr. Each data point represents a triplicate sample from two independent experiments. Results are plotted as a response ratio (normalized against untreated values).

The GR transactivation potency for DEX, FA and TA has never been directly compared in one cell type. Moreover, a wide range of GR transactivation potency values have been reported in the literature for each of these three GCs [35]. Therefore, GR transactivation potency was assessed in the GeneBLAzer β-lactamase reporter gene technology in MMTV-bla HeLa CellSensor^® ^cells (Invitrogen). Attempts to establish a robust and reproducible GeneBLAzer assay using primary human TM cells failed, and HeLa cells seemed a reasonable surrogate since the levels of all GRα and GRβ isoforms in these cells were comparable to those in TM 86 and TM 93 cells (Figure 1). HeLa cells were treated with various concentrations of GC for 5 hr. Figure 2 shows that all three GCs induced an increase in GR transactivation activity in a dose-dependent manner. The EC_50 _(concentration causing a half-maximal increase in GR transactivation) values for DEX, FA, and TA, were 3.02 ± 0.02, 0.67 ± 0.10, and 1.29 ± 0.28 nM, respectively. These data suggest that all three GCs are roughly equivalent at stimulating GR transcriptional activity. The expression of multiple GRα isoforms in HeLa cells has been previously reported [20] and confirmed in our study. Assuming that different GRα isoforms might exhibit different transcriptional activities in response to each GC, the observed GR-mediated transactivation function is the aggregate response generated by multiple GR isoforms in these cells.

### GRα and GRβ isoform expression in TM 86 and TM 93 cells

Levels of total GRα and total GRβ proteins have been previously reported in human TM by competitive binding assay [18] or Western blot [17]. However, to our knowledge, the expression of GRα and GRβ translational isoforms in human TM cells has never been determined. The distribution of four GRα isoforms, GRα-A, GRα-B, GRα-C, and GRα-D was assessed in TM 86 and TM 93 total cell lysates by Western blot using a specific anti-GRα antibody. Western blots showed that both TM 86 and TM 93 expressed all four known GRα isoforms (Figure 1A). Higher levels of GRα-C and GRα-D isoforms were observed compared to GRα-A and GRα-B. The additional bands in between GRα-C and GRα-D isoforms may be degradation products or cell-specific isoforms. The distribution of four GRβ isoforms, GRβ-A, GRβ-B, GRβ-C, and GRβ-D was also determined in TM 86 and TM 93 total cell lysates by Western blot using a specific anti-GRβ antibody. Figure 1B shows that both TM 86 and TM 93 expressed all four known GRβ isoforms. The level of GRβ-D isoform was lower when compared to levels of GRβ-A, GRβ-B, and GRβ-C in both TM 86 and TM 93 cells. Moreover, TM 93 expressed lower levels of GRβ-D as compared to that in TM 86. The additional bands in between GRβ-C and GRβ-D isoforms may be degradation products or cell-specific isoforms.

### Gene expression profiles for DEX, FA, and TA in TM 86 and TM 93 cells

**Figure 3:**
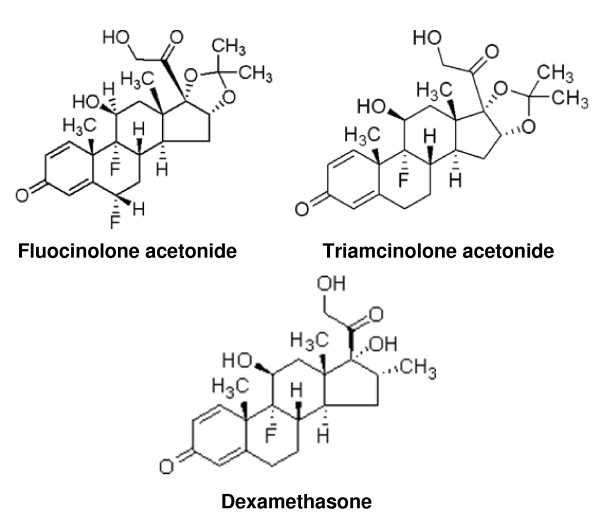
**Structures of dexamethasone, fluocinolone acetonide, and triamcinolone acetonide**.

The chemical structures of DEX, FA, and TA are shown in Figure 3. To determine the biological impact of GC structure on GR transcription, primary TM 86 and TM 93 cells were treated with DEX, FA, TA, or a vehicle control and total RNA was isolated 24 hr later followed by gene expression profiling using Agilent Whole Human Genome Microarrays. The saturating concentration of 1 μM for each GC was chosen based on the GeneBLAzer reporter gene assay results (Figure 2) and human clinical reports showing ocular tissue concentrations for DEX or TA >1 μM following local administration [36,37]. Three biological replicates were analyzed using independent microarrays for each drug treatment that were then directly compared to vehicle controls (0.1% DMSO).

**Figure 4:**
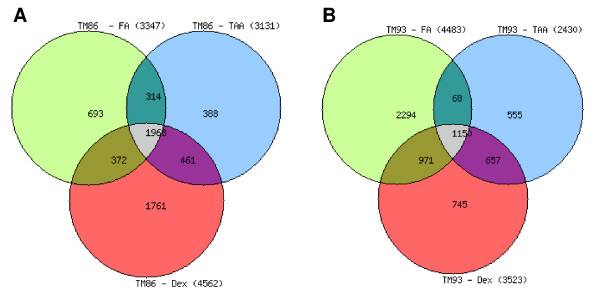
**Venn diagrams showing the distribution and overlap of differentially expressed genes in TM 86 (A) and TM 93 (B) cells**. TM cells were treated with dexamethasone, fluocinolone acetonide, or triamcinolone acetonide at 1 μM for 24 hr. The two criteria for the identification of significantly different gene expression were log ratio *p *< 0.001 and log (10) intensity measurement >-1.8.

Since TM 86 and TM 93 cells were isolated from donors of different ages, all subsequent microarray data analysis were performed for each TM cell type, separately [for complete gene lists see additional file 1]. There were 1968 genes regulated by all three GCs in TM 86 cells (Figure 4A). However, the total number of genes uniquely regulated by each GC varies: 1761, 693, or 388 for DEX, FA, or TA, respectively, suggesting a potential transcriptional mechanism underlying the different cellular responses to each of the GCs (Figure 4A). In TM 93 cells, 1150 genes were regulated commonly by all three GCs (Figure 4B). Each of the GCs also regulated a unique set of genes in TM 93 cells. There were 745, 2294, or 555 genes regulated uniquely by DEX, FA, or TA, respectively (Figure 4B).

Table 2 summarizes the top 24 genes regulated by DEX, FA or TA in TM 86 cells based on the magnitude of fold change. These genes included RGC32, ANGPTL7, MYOC, FKBP5, SAA1, MXRA5, PER3 and AQP1. Interestingly, OCA2 and PF4 were found to be uniquely upregulated by 70.2- or 3.1-fold in response to DEX or FA, respectively. Table 3 shows the 24 genes most highly upregulated by DEX, FA or TA in TM 93 cells. The list of genes included OCA2, SAA1, ANGPTL7, FKBP5, MYOC, AQP1, MXRA5 and PER3. Similar to TM 86, SLP1 was found to be uniquely upregulated by 18.0-fold in response to FA.

**Table 2:** Top 24 differentially regulated genes in response to DEX, FA, or TA treatment in TM 86 cell line.

**Gene Symbol**	**Gene Name**	**GenBank Accession Number**	**DEX** **FC**	**FA** **FC**	**TA** **FC**
UPREGULATED					

*RGC32*	Response gene to complement 32	NM_014059	>100	>100	>100
*OCA2*	oculocutaneous albinism II	NM_000275	70.2	NS	NS
*SAA1*	Serum amyloid A1	NM_000331	30.9	34.6	35.3
*ANGPTL7*	Angiopoietin-like 7	NM_021146	24.8	16.0	26.5
*ITGA10*	Integrin, alpha 10	NM_003637	22.4	25.1	17.7
*LSP1*	clone pp9372 unknown mRNA	AF289610	20.9	14.9	15.4
*KCNB1*	Potassium voltage-gated channel, Shab-related subfamily, member 1	NM_004975	13.6	20.1	19.9
*FKBP5*	FK506 binding protein 5	NM_004117	12.9	13.8	12.5
*KLF15*	Kruppel-like factor 15	NM_014079	12.5	11.5	11.1
*ANGPTL4*	Angiopoietin-like 4	NM_139314	7.0	9.6	7.9
*MYOC*	Myocilin, trabecular meshwork inducible glucocorticoid response	NM_000261	7.5	7.4	6.7
*ZBTB16*	Zinc finger and BTB domain containing 16	NM_006006	7.5	8.7	8.1
*PF4*	platelet factor 4 (chemokine (C-X-C motif) ligand 4)	NM_002619	NS	3.2	NS

DOWNREGULATED					

*KAL1*	Kallmann syndrome 1 sequence	NM_000216	-5.8	-5.6	-6.7
*GABRB1*	Gamma-aminobutyric acid (GABA) A receptor, beta 1	NM_000812	-6.9	-4.0	-4.9
*MXRA5*	Matrix-remodelling associated 5	NM_015419	-6.0	-4.3	-5.4
*PER3*	Period homolog 3 (Drosophila)	NM_016831	-5.1	-5.3	-7.0
*AQP1*	Aquaporin 1 (Colton blood group)	NM_198098	-5.5	-4.4	-5.1
*PCDH19*	Protocadherin 19	NM_020766	-4.7	-5.1	-5.0
*RASL11B*	RAS-like, family 11, member B	NM_023940	-4.0	-4.9	-4.0
*KIF4A*	Kinesin family member 4A	NM_012310	-4.0	-3.6	-4.1
*CXADR*	Coxsackie virus and adenovirus receptor	NM_001338	-4.7	-3.4	-4.8
*CCRL1*	Chemokine (C-C motif) receptor-like 1	NM_178445	-3.8	-4.7	-3.4
*ENST00000344214*	Unknown	Unknown	NS	NS	-3.1

FC: fold change; NS: not significant

The genes are listed according to their rank order fold-change.

**Table 3:** Top 24 differentially regulated genes in response to DEX, FA, or TA treatment in TM 93 cell line.

**Gene Symbol**	**Gene Name**	**GenBank Accession Number**	**DEX** **FC**	**FA** **FC**	**TA** **FC**
UPREGULATED					
*OCA2*	oculocutaneous albinism II	NM_000275	>100	98.0	>100
*ANGPTL7*	Angiopoietin-like 7	NM_021146	18.8	13.0	20.5
*RGC32*	Response gene to complement 32	NM_014059	18.8	11.0	19.9
*LSP1*	clone pp9372 unknown mRNA	AF289610	NS	18.0	NS
*SAA1*	Serum amyloid A1	NM_000331	10.7	8.3	11.9
*KCNB1*	Potassium voltage-gated channel, Shab-related subfamily, member 1	NM_004975	9.3	5.2	11.3
*ITGA10*	Integrin, alpha 10	NM_003637	8.1	9.1	5.6
*ANGPTL4*	Angiopoietin-like 4	NM_139314	6.5	9.1	5.3
*FKBP5*	FK506 binding protein 5	NM_004117	5.9	6.8	5.8
*PF4*	platelet factor 4 (chemokine (C-X-C motif) ligand 4)	NM_002619	5.2	6.5	4.4
*ZBTB16*	Zinc finger and BTB domain containing 16	NM_006006	5.4	4.9	5.5
*KLF15*	Kruppel-like factor 15	NM_014079	4.2	3.6	3.9
*MYOC*	Myocilin, trabecular meshwork inducible glucocorticoid response	NM_000261	2.5	2.7	2.6

DOWNREGULATED					

*ENST00000344214*	Unknown	Unknown	-5.6	-6.3	NS
*KAL1*	Kallmann syndrome 1 sequence	NM_000216	-2.9	-3.0	-3.3
*GABRB1*	Gamma-aminobutyric acid (GABA) A receptor, beta 1	NM_000812	-2.5	-2.2	-2.6
*AQP1*	Aquaporin 1 (Colton blood group)	NM_198098	-2.8	-3.2	-2.8
*MXRA5*	Matrix-remodelling associated 5	NM_015419	-3.9	-4.5	-4.5
*PCDH19*	Protocadherin 19	NM_020766	-2.9	-3.9	-3.3
*PER3*	Period homolog 3 (Drosophila)	NM_016831	-2.8	-3.6	-3.8
*CCRL1*	Chemokine (C-C motif) receptor-like 1	NM_178445	-2.4	-2.5	-2.1
*KIF4A*	Kinesin family member 4A	NM_012310	-2.3	-2.2	-2.3
*CXADR*	Coxsackie virus and adenovirus receptor	NM_001338	-2.2	-1.8	-2.6
*RASL11B*	RAS-like, family 11, member B	NM_023940	-1.5	NS	NS

FC: fold change; NS: not significant

The genes are listed according to their rank order fold-change.

### Gene function analysis

**Figure 5:**
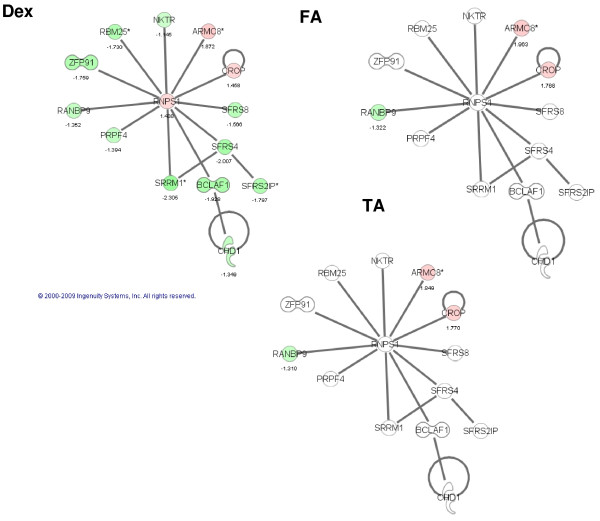
**The most prominently affected gene network in response to DEX in TM 86 cells**. Network was classified as: *RNA-Post-Transcriptional Modification, Antigen Presentation, Immune cell Trafficking*. Regulation of this network in TM 86 cells is compared across treatment with DEX, FA or TA. Pathway contains RNA binding protein S1 (RNPS1), which occupies a central position in this network. Red: induction; green: repression; white: unaffected; color intensity correlates with fold change; number: fold change. The diagram was obtained from Ingenuity Pathway Analysis (Ingenuity^® ^Systems).

**Figure 6:**
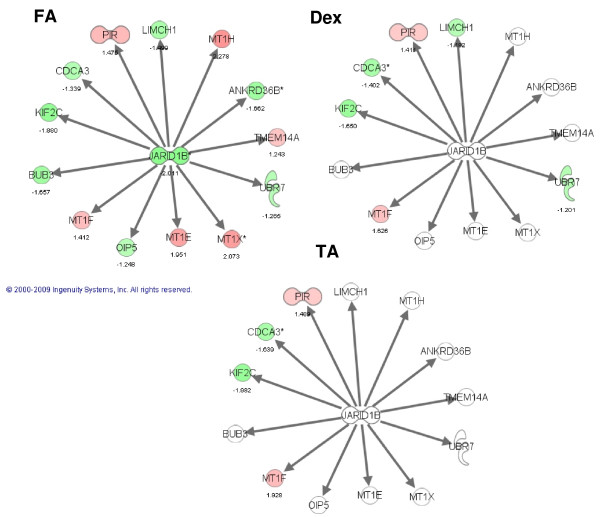
**The most prominently affected gene network in response to FA in TM 93 cells**. Network was classified as: *Cell Cycle, Cell-mediated Immune Response, Cellular Assembly and Organization*. Regulation of this network in TM 93 cells is compared across treatment with DEX, FA or TA. Pathway contains jumonji, AT rich interactive domain 1B transcriptional repressor (JARID1B), which occupies a central position in this network. Red: induction; green: repression; white: unaffected; color intensity correlates with fold change; number: fold change. The diagram was obtained from Ingenuity Pathway Analysis (Ingenuity^® ^Systems).

Using Ingenuity Pathway Analysis, we identified networks that were significantly enriched using the lists of differentially expressed genes. This analysis confirmed the unique regulation of a functional subset of genes by each of the GCs in TM 86 and TM 93 cells. For example, in TM 86, DEX significantly regulated a network centered around the RNA binding protein S1 or RNPS1 (Figure 5), whereas FA significantly modulated a GR-dependent network (data not shown). In TM 93, DEX significantly affected a network centered around a histone methyltransferase known as suppressor of zeste 12 homolog or SUZ12 (data not shown), whereas FA regulated a transcriptional repressor referred to as jumonji, AT rich interactive domain 1B or JARID1B-based network (Figure 6).

Using the High-Throughput GoMiner integrative gene ontology tool, the enrichment of biological themes from the differentially expressed transcript lists in response to each of the GCs was also evaluated in both TM 86 and TM 93 cell lines [for complete biological processes lists see additional files 2, 3, 4, 5, 6, 7, 8 and 9]. This analysis confirmed again that each of the GCs can generate both a common and unique pattern of gene expression that is associated with specific biological processes. Results showed that in TM 86 cells, DEX significantly regulated cellular component organization and biogenesis, whereas FA and TA regulated actin filament-based process and mitotic cell cycle, respectively. In TM 93 cells, DEX significantly regulated membrane lipid metabolic process, whereas FA and TA regulated biopolymer metabolic process and smooth muscle cell proliferation, respectively.

## Discussion

The goals of this study were to measure GR potency for three GCs used to treat inflammatory eye disease, examine the expression of GRα and GRβ translational isoforms in human TM cells, and to assess changes in mRNA levels in TM cells treated with DEX, FA or TA. The findings of this study demonstrate that TM cells *in vitro *express all GRα and GRβ N-terminal isoforms, and that GCs with dissimilar structures but similar GR potency are capable of regulating common and unique subsets of genes and biological networks that appear to be cell-lineage dependent.

Before characterizing GC transcript responses in TM cells, we deemed it important to directly measure and compare the GR binding affinity for DEX, FA, and TA, and their potency in a GR-mediated transactivation assay. The GR binding affinities for all three GCs were in the low nM range and were considered roughly equivalent. Importantly, the GR binding affinity of 5 nM for DEX reported here in IM-9 B-lymphoblastoid cells is identical to that reported in primary human TM cells [18]. The EC_50 _values for DEX, FA, and TA in the GR-mediated HELA cell transactivation assay were also in the low nM range. These data support the notion that these three GCs are roughly equivalent at stimulating GR-mediated transcriptional activity [16]. The relationship between receptor potency and GR translational isoform expression is to date poorly understood. The expression of GRα isoforms in HeLa cells has been previously reported [20] and confirmed in our study, however, the distribution of GRα isoforms in human IM-9 cells used in the GR binding affinity assay is unknown. Assuming that different GRα isoforms may exhibit different binding affinities for each GC, the observed GR binding affinity or transactivation potency is the aggregate affinity or potency generated by multiple GR isoforms in these cells. To avoid possible subtle but significant differences in GC potencies in TM cells, we chose a saturating GC dose of 1 μM in subsequent gene regulation studies.

Recently, Lu and Cidlowski showed distinct tissue-specific distribution patterns of N-terminal GRα isoforms in mice and rat tissues, and suggested that unique GRα transcriptional activities and distinct tissue-specific distribution patterns could provide a novel mechanism for tissue-specific glucocorticoid responses [20]. Total GRα and GRβ protein expression was previously reported in human TM by a competitive binding assay [18] or by Western blot [17]. Our results show that TM 86 and TM 93 cells express detectable levels of all known GRα or GRβ isoforms (A-D). To our knowledge, this is the first report of GRα and GRβ translational isoforms in human TM cells. Although the relationship between GC-mediated biological responses and the distribution of GRα and GRβ isoforms in TM cells is currently undefined, it is tempting to speculate that GRα-C may play an important role in TM cells since it was shown that this isoform has the highest relative transcriptional activity in response to DEX [20].

Gene expression profiling studies using Agilent microarray revealed that the two primary TM cell types exhibited significantly different global gene expression profiles. This observation may be explained by a higher level of GRβ-D isoform expression in TM 86 compared to TM 93 cells. Alternatively, the fact that TM 86 and TM 93 cells were isolated from an infant and an adult, respectively, may account for the differences in gene expression patterns between the two cell lines. It is also possible that inherent differences between individuals may reflect differences in gene expression profiles.

To test the hypothesis that each GC generates subsets of common and unique genes, we compared the global gene expression profiles of two different TM cell types in response to 24 hr treatment with a saturating GC concentration. This is the first microarray study that compares the effects of three clinically-relevant GCs at equimolar concentrations on the transcriptome of TM cells. Our study shows that many genes commonly regulated by DEX, FA and TA in TM 86 and TM 93 cells were also regulated by DEX in published studies in primary TM cells [8-11]. These genes included MYOC, FKBP5, SAA1, SAA2, ZBTB16, ANGPTL7, CXADR and PER3. The size of this common subset of genes was steroid and cell-dependent, and accounted for 16%-53% of all steroid-regulated genes in TM86 cells and 33%-67% in TM93 cells.

Our results also showed that all three GCs regulated, in a cell lineage-dependent manner, unique sets of genes involved in a specific biological process or functional network. The present study closely confirms recently published findings by Fan et al who demonstrated that TA and DEX were capable of regulating common as well as unique subsets of genes in TM cells [13]. The Fan et al results showed that GC-regulated genes were associated with multiple cell functions including acute-phase response, cell adhesion, and cell cycle and growth. Similar observations were obtained by Guo et al who showed that different peroxisome proliferator-activated receptor α or γ agonists were capable of generating common and unique gene expression subsets in rodent primary hepatocytes [38,39]. In the present study, gene function analysis showed that in TM 86 cells, DEX significantly regulated a network centered around the RNA binding protein S1 or RNPS1, whereas FA significantly modulated a GR-dependent network. In TM 93, DEX significantly affected histone methyltransferase or SUZ12 network, whereas FA regulated transcriptional repressor or JARID1B-based network.

The functional and potential clinical significance of these GC-dependent pharmacologic responses requires further investigation. These responses are likely GC dose and duration of exposure-dependent, and the results suggest a very complex mechanism that is initiated by GC-GRα interactions. For example, in addition to unique GC-dependent gene subsets, specific genes such as OCA2 and SLP1 were uniquely regulated by only one GC in one cell line and regulated by all three GCs in the other cell line. These observations suggest that in a specific cell line DEX, FA, or TA generate a unique ligand-GR binding conformation, a unique GR-DNA binding domain conformation, and the subsequent recruitment of distinct co-repressors or co-activators. There may also be GC-dependent remodeling of the chromatin at the promoter of GR target genes.

Finally, the microarray results presented here further support the findings by Schaaf and Cidlowski that show GC analogs with subtly different chemical structures are capable of generating unique patterns of GC-GRα nuclear distribution and mobility [21]. Our microarray data strongly suggest that the GC-mediated GR nuclear distribution patterns ultimately regulate common and unique subsets of genes and presumably generate unique cellular responses. The clinical impact of pharmacologically-distinct and defined glucocorticoid agonists on inflammatory diseases, including those of the eye, remains fertile ground for future research.

## Conclusion

A primary goal of this study was to determine if glucocorticoids with similar potencies but subtly different chemical structures can generate both common and unique subsets of genes in trabecular meshwork cells. This hypothesis was proposed by Schaaf and Cidlowski from studies showing that GC chemical structure directly influenced GC/GRα nuclear distribution and mobility [21]. In surrogate cell types, the GR binding affinity and GR-mediated transactivation potencies for DEX, FA, or TA in are similar (1 - 5 nM), and represent the aggregate affinity or transactivation potency generated by multiple GRα and GRβ isoforms. All four known GRα and GRβ translational isoforms (A-D) were expressed in TM 86 and TM 93 cells. The physiologic necessity and functional role for multiple GRα isoforms in TM cells requires further study, however, gene expression analysis revealed that 1,968 and 1,150 genes were regulated by DEX, FA, and TA in TM 86 and TM 93 cells, respectively. Many of these genes, including MYOC, FKBP5, SAA1, ZBTB16 and ANGPTL7 were previously reported to be regulated by DEX or TA in primary human TM cells. In addition to sets of genes shared by all three GCs, each GC also regulated a unique subset of genes in both TM cell lines. In TM 86, DEX significantly regulated transcripts associated with RNA post-transcriptional modifications, whereas FA and TA modulated genes associated with lipid metabolism and cell morphology, respectively. In TM 93 cells, DEX significantly regulated genes related to histone methylation, whereas FA and TA regulated genes related to cell cycle and cell adhesion, respectively. These observations support the hypothesis that DEX, FA, or TA are capable of generating common and unique gene subsets via a unique conformation of the GC-GRα complex, which ultimately results in the binding of GRα to specific promoters on target genes. These unique GC-mediated unique molecular effects are likely time, dose, cell-type, and cell-lineage dependent.

## Competing interests

Although AN and JLE are current employees at Allergan Inc., we are not discussing or using any current or future commercial products.

## Authors' contributions

AN conducted Western blot experiments, prepared TM cell samples for microarray studies, conducted the IPA analysis of microarray data, coordinated the Allergan business contracts with Cerep, Invitrogen, and Cogenics, and wrote the manuscript. EKL performed the analysis of microarray data. WDS provided the primary human trabecular meshwork cells used in this study and consulted on use of primary cultures of TM cells as model for TM tissues. JLE, EKL and WDS helped writing the manuscript. All authors read and approved the final manuscript.

## Pre-publication history

The pre-publication history for this paper can be accessed here:



## Supplementary Material

Additional file 1 **Comparison of lists of differentially expressed genes in response to DEX, FA, and TA in TM 86 and TM 93**. These lists show, for TM 86 and TM 93, the genes that were differentially regulated in response to a single treatment (DEX, FA, or TA), as well as the genes commonly expressed across multiple treatments. Click here for file

Additional file 2 **The "change" list of enriched gene ontological processes in response to DEX in TM 86**. A list of biological processes (and associated statistical enrichment values) representing the entire list of transcripts and negating the directionality of the change. The list was sorted by *p*-value. Click here for file

Additional file 3 **The "change" list of enriched gene ontological processes in response to FA in TM 86**. A list of biological processes (and associated statistical enrichment values) representing the entire list of transcripts and negating the directionality of the change. The list was sorted by *p*-value. Click here for file

Additional file 4 **The "change" list of enriched gene ontological processes in response to TA in TM 86**. A list of biological processes (and associated statistical enrichment values) representing the entire list of transcripts and negating the directionality of the change. The list was sorted by *p*-value. Click here for file

Additional file 5 **The "change" list of enriched gene ontological processes in response to DEX, FA and TA in TM 86**. A list of biological processes (and associated statistical enrichment values) representing the entire list of transcripts and negating the directionality of the change. The list was sorted by *p*-value. Click here for file

Additional file 6 **The "change" list of enriched gene ontological processes in response to DEX in TM 93**. A list of biological processes (and associated statistical enrichment values) representing the entire list of transcripts and negating the directionality of the change. The list was sorted by *p*-value. Click here for file

Additional file 7 **The "change" list of enriched gene ontological processes in response to FA in TM 93**. A list of biological processes (and associated statistical enrichment values) representing the entire list of transcripts and negating the directionality of the change. The list was sorted by *p*-value. Click here for file

Additional file 8 **The "change" list of enriched gene ontological processes in response to TA in TM 93**. A list of biological processes (and associated statistical enrichment values) representing the entire list of transcripts and negating the directionality of the change. The list was sorted by *p*-value. Click here for file

Additional file 9 **The "change" list of enriched gene ontological processes in response to DEX, FA and TA in TM 93**. A list of biological processes (and associated statistical enrichment values) representing the entire list of transcripts and negating the directionality of the change. The list was sorted by *p*-value. Click here for file
